# Antifungal Susceptibility and *Cyp51A* Gene Variation Analysis of *Aspergillus fumigatus* Isolated from Soils in Tea-Growing Areas of Guizhou, China

**DOI:** 10.3390/microorganisms14081704

**Published:** 2026-08-04

**Authors:** Duanyong Zhou, Yixian Liu, Mingyue Wang, Cai Yang, Ying Zhang, Jianping Xu

**Affiliations:** 1 Key Laboratory of Agricultural Microbial Resources Development and Utilization of Qianxinan Prefecture, Minzu Normal University of Xingyi, Xingyi 562400, China; zhouduanyong@xynun.edu.cn (D.Z.);; 2 Research Centre of Plant Protection, Yunnan Institute of Tropical Crops, Jinghong 666101, China; 3 State Key Laboratory for Conservation and Utilization of Bio-Resources in Yunnan, Yunnan University, Kunming 650500, China; 4 Department of Biology, McMaster University, Hamilton, ON L8S 4K1, Canada

**Keywords:** triazole susceptibility, minimum inhibitory concentration, geographic populations, allele distribution, non-resistance-associated mutations

## Abstract

*Aspergillus fumigatus* is the predominant pathogenic fungus responsible for aspergillosis. In recent years, the global detection rate of azole-resistant *A. fumigatus* has continuously increased, and the extensive application of agricultural azole fungicides has been recognized as a crucial driving factor for the emergence and spread of resistance mutations in environmental *A. fumigatus*. Previous investigations conducted by our research team in karst vegetable fields of Guizhou Province revealed that the triazole resistance rate of local *A. fumigatus* was only 0.49%, which was markedly lower than those reported in most previous studies in China and outside of China. To supplement the prevalence data of azole resistance across different habitats in this region, a total of 191 environmental *A. fumigatus* strains were isolated from nine tea plantations across Guizhou. In this study, two clinically prevalent azole drugs, itraconazole and voriconazole, were used for antifungal susceptibility testing, and the triazole target gene *cyp51A* of all isolates was sequenced and analyzed. Antifungal susceptibility results demonstrated that the MIC ranges of the tea plantation *A. fumigatus* population were 0.015–0.5 μg/mL for itraconazole and 0.031–0.25 μg/mL for voriconazole, with no evidence of triazole resistance. Genetic analysis identified ten different gene mutations among 29 isolates, all of which were classified as non-resistance-associated mutations. Among these mutations, four were synonymous mutations, including 267G→A, 540G→A, 1074A→G, and 1362T→C, while six were non-synonymous mutations, including 137T→A, 514A→G, 743A→C, 744T→A, 765C→G, and 1279G→A. These non-synonymous mutations resulted in five amino acid substitutions in 25 strains, namely F46Y, M172V, N248T/K, D255E, and E427K. The N248T/K mutation exhibited the highest mutational frequency of 0.1309 (25/191) and was distributed across all nine sampling sites. Correlation analyses indicated that no significant correlations were observed between all detected variant loci and MICs of isolates to itraconazole and voriconazole. Phylogenetic analysis revealed that the six sequence types of *cyp51A* in Guizhou tea plantations were broadly intermixed with those from other parts of China and outside of China. We discussed the implications of these results in the management of antifungal resistance.

## 1. Introduction

*Aspergillus fumigatus* is a ubiquitous saprophytic and opportunistic pathogenic fungus with strong environmental adaptability, which widely colonizes soil, air, plant rhizosphere, and organic-rich habitats such as humus and compost [1,2,3,4]. The conidia of *A. fumigatus* are widely transmitted via the air [5,6]. In immunocompromised individuals, inhalation of airborne conidia readily causes invasive aspergillosis, with an overall clinical mortality rate ranging from 40% to 90% [7,8,9]. Clinical therapeutic and prophylactic options for *A. fumigatus*-associated aspergillosis are relatively scarce. Azole antifungal agents, including itraconazole, posaconazole and voriconazole, serve as the first-line core medications for the clinical prevention and treatment of such fungal infections [10,11].

In recent years, a growing number of studies have reported the isolation of azole-resistant *A. fumigatus* (ARAF) strains from patients with aspergillosis [12,13]. While azole resistance can be induced during antifungal therapy [9,14,15], the widespread application of agricultural azole fungicides for crop protection has now been firmly linked to the emergence of azole resistance in environmental *A. fumigatus* populations [9,16,17]. The *cyp51A* gene encodes the target enzyme lanosterol 14α-demethylase, and mutations in this gene represent the predominant molecular mechanism of azole resistance in clinical and environmental *A. fumigatus* isolates [18,19,20,21]. Accumulated evidence has demonstrated that amino acid substitutions at the G54W/E/R/V, Y121F, G138C, P216L, F219C, M220K/T, A284T, Y431C, G432A, G434C and G448S loci serve as the predominant mutations closely associated with azole resistance in this pathogen by reducing the binding affinity of triazoles to the drug target CYP51A [17,22,23,24,25,26,27,28].

Guizhou Province is in southwest China, with an extremely high proportion of karst landforms, mountains and hills. It has a fragile ecological environment and is widely distributed with underground karst caves, which significantly increases the difficulty and cost of local transportation construction. Restricted by such geographical conditions, Guizhou has long remained in a relatively closed and isolated state in history [29,30,31]. Our research team previously conducted a surveillance on the prevalence of ARAF in soil samples from vegetable gardens of farmers at 9 sites in Guizhou Province. The results showed that the frequency of azole resistance in that population of *A. fumigatus* was only 0.49% (1/206), among the lowest reported so far in the global environmental populations of *A. fumigatus* [4].

As a core green tea-producing area in China, Guizhou has prominent advantages in the development of the tea industry. In 2025, the tea-growing area of the province reached 4700 square kilometers, the tea output reached 3.26 × 10^7^ kg, and the comprehensive output value of the tea industry exceeded 14.6 billion US dollars. It has become a distinctive and advantageous pillar industry in the region, effectively driving farmers’ employment and increasing their income. To further understand the epidemiological characteristics of ARAF in environmental samples in Guizhou, this study conducted extensive sampling in tea-growing areas at nine distinct geographic locations in Guizhou Province, determined the drug susceptibility of the tested strains to two common triazole drugs used for clinical treatment of aspergillosis—itraconazole and voriconazole—and performed DNA sequencing and sequence alignment analysis of the triazole target gene *cyp51A* for all strains. The objectives of this study are: (1) to clarify the prevalence and distribution characteristics of ARAF in tea-growing area soils in Guizhou; (2) to explore the genetic variation patterns of the azole target gene *cyp51A*; (3) to investigate the correlation between *cyp51A* sequence variations and triazole minimum inhibitory concentration (MIC).

## 2. Materials and Methods

### 2.1. Soil Sampling, Isolation and Identification of A. fumigatus

**Figure 1:**
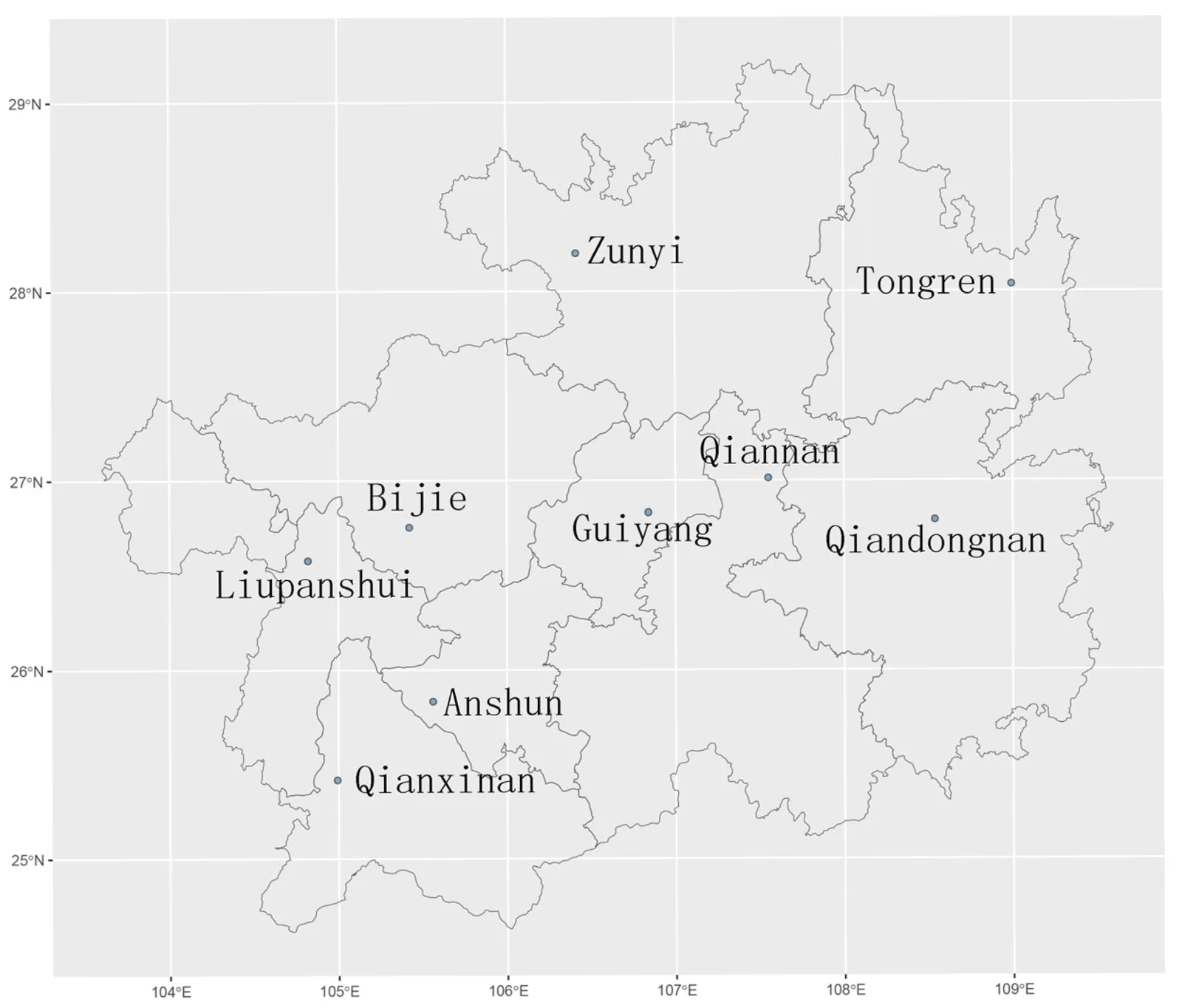
Geographical distribution of *A. fumigatus* samples included in this study. Soil samples from nine tea-plantations in nine different administrative regions within Guizhou province in southwestern China were analyzed. The locations of the plantations are shown as black dots. Each dot corresponds to a nominal 100 m^2^ plot from which 100 soil samples were collected.

Soil sampling was carried out across nine tea plantations in Guizhou from 29 August to 1 September 2023. At each of the nine tea plantations, 100 topsoil samples weighing roughly 10 g were taken at a depth of 0 to 5 cm, with one-meter intervals between adjacent sampling points [32]. Geographic details of sampling sites are illustrated in Figure 1. Each soil sample was stored in a separate sterile zipper bag. Isolation of *A. fumigatus* was performed following the protocol described previously [4,17]. Briefly, 1ml of Sabouraud Dextrose (SD) broth supplemented with chloramphenicol (50 µg/mL) was thoroughly mixed with 0.1 to 0.2 g of soil and incubated for 2 days at 50 °C to select for *A. fumigatus* growth. After incubation, mycelia that had grown at the surface to air boundary were transferred to an SD agar plate and again incubated for 2 days at 50 °C. Conidia from green suede-like growth characteristic of *A. fumigatus* were harvested using a wooden applicator stick and streaked onto SD agar to obtain individual colonies. After incubation for 2 days at 37 °C, microscopy was used to identify uniseriate conidiophore morphology and the conidia from these colonies were harvested using 750 µL of 30% sterile glycerol in water and stored at −80 °C. Initial and final identification of the strains was conducted according to the methods reported in our prior studies [32,33]. We aimed to obtain >20 isolates of *A. fumigatus* from each geographic region for analyes.

### 2.2. Triazole Susceptibility of Testing

Two clinical azole drugs (itraconazole and voriconazole) commonly used for the treatment of aspergillosis were used to test the susceptibility of *A. fumigatus* isolated in this study following the methods described in the CLSI M38-A3 [34]. Briefly, the broth microdilution method used 96-well microtiter plates where each well containing 100 μL of serially (twofold) diluted drug concentrations was inoculated with 100 μL of the twofold RPMI-1640 medium containing 2 × 10^3^ conidia. The final volume in each well was 200 μL. These plates were incubated at 35 °C and examined at 48 h for MIC determination. Strains *Candida parapsilosis* ATCC22019 and *Candida krusei* ATCC6258 were used as references. The minimum inhibitory concentration (MIC) is defined as the lowest drug concentration that achieves complete (100%) inhibition of mycelial growth of the tested strains by visual observation. GM refers to geometric mean, the average drug concentration calculated logarithmically. GM provides a stable central tendency of susceptibility for the tested population without being skewed by extreme high or low values. MIC_50_ refers to the lowest concentration capable of inhibiting the growth of 50% of the tested strain population; MIC_90_ represents the lowest concentration that inhibits the growth of 90% of the tested strain population.

### 2.3. Sequencing of the cyp51A Gene

Genomic DNA of each of the 191 isolates was extracted following protocols described previously [26]. The extracted genomic DNA was used as a template for targeted amplification of the target gene *cyp51A* using primer pair A7 (5′-TCATATGTTGCTCAGCGG-3′) and P450-A2 (5′-CTGTCTCACTTGGATGTG-3′) [26]. The PCR amplification protocol was performed as follows: initial denaturation at 95 °C for 3 min, followed by 35 cycles of denaturation at 95 °C for 30 s, annealing at 57 °C for 30 s, and extension at 72 °C for 2 min, with a final extension at 72 °C for 10 min. The fungal DNA extraction kit, PCR kit, and PCR product purification kit were all purchased from Tsingke Biotechnology Co., Ltd. (Beijing, China). After agarose gel electrophoresis for confirmation of PCR amplification success, PCR products were sequenced at Tsingke Biotechnology Co., Ltd. for bidirectional Sanger sequencing.

### 2.4. cyp51A Gene Mutation Analysis and Phylogenetic Analysis

Mutations of the *cyp51A* gene and its promoter region were identified by comparing with the reference sequence of a wild-type azole-sensitive *A. fumigatus* strain under the accession number AF338659 in GenBank [17,35,36]. The polymorphism of the *cyp51A* gene was analyzed using DnaSP software v5 [37]. Sequence alignment and phylogenetic tree construction of representative sequences were performed using MEGA 6.0 software [38]. To investigate whether the *cyp51A* sequences in our study were unique to Guizhou and evolutionarily clustered together, we compared our *cyp51A* gene sequences in *A. fumigatus* with those from diverse global geographical origins. Here, a total of 1303 *cyp51A* gene sequences of *A. fumigatus* were retrieved from the NCBI database for comparison. These sequences originated from 19 countries across the globe: China contributed 802 sequences, followed by France (195), Japan (71), Italy (61), the United Kingdom (36), the Republic of Korea (26), India (24), Portugal (19), Austria (18), Spain (14), Brazil (13), the United States (9), Canada (4), Australia (4), Kuwait (3), Peru (3), Denmark (2), and the Netherlands and Colombia each supplied one sequence. Phylogenetic analysis of the total dataset used the MEGA 6.0 software [38].

### 2.5. Statistical Analysis

Box plots were generated to illustrate the distribution of minimum inhibitory concentrations (MICs) of each antifungal agent across different geographical populations. The Kruskal–Wallis test was used to analyze differences in MIC values among geographical groups. All box plot construction and statistical analyses were performed using GraphPad Prism 10.6.0 (GraphPad Software, San Diego, CA, USA). IBM SPSS Statistics 22.0 was used to analyze the potential correlations between *cyp51A* gene mutation sites and antifungal MIC values.

## 3. Results

### 3.1. Isolation and Susceptibility of A. fumigatus Isolates

**Figure 2:**
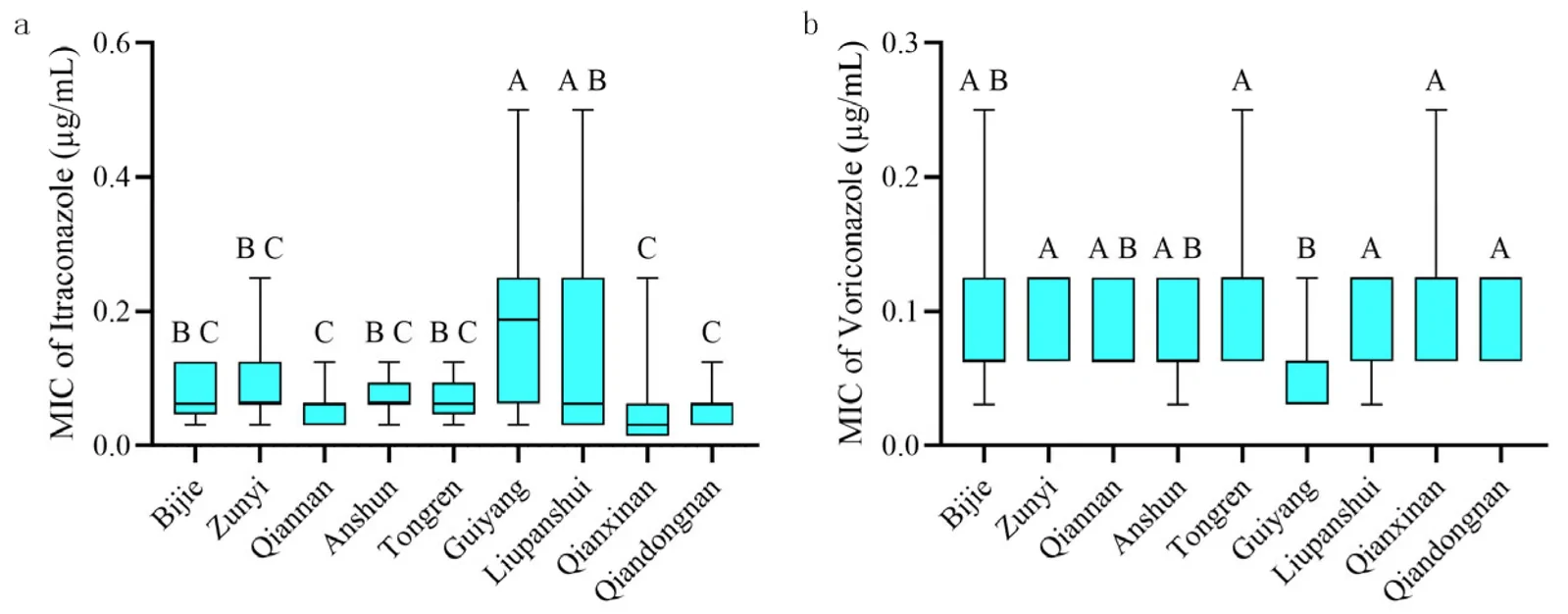
Boxplot comparison of minimum inhibitory concentration (MIC) values (Y-axis) of *A. fumigatus* against itraconazole (**a**) and voriconazole (**b**) among different geographical populations (X-axis). Populations with different letters (A, B, C) indicate statistically significant differences in their MIC distributions (*p* < 0.05).

In this study, 191 *A. fumigatus* strains were isolated and identified from 900 soil samples collected from 9 tea-growing areas in Guizhou Province (Figure 1). Among them, 22 strains were isolated from Guiyang and Qiandongnan respectively, and 21 strains were isolated from each of the other seven sampling sites. The isolation frequencies of *A. fumigatus* at each sampling site ranged from 20% to 21%, with similar isolation frequencies across sites. Antifungal susceptibility testing showed that all 191 *A. fumigatus* isolates in this study were sensitive to azole drugs. For itraconazole, the MIC values ranged from 0.015 to 0.5 μg/mL, and the geometric mean MIC (GM-MIC) was 0.067 μg/mL, with an MIC_50_ of 0.063 μg/mL and an MIC_90_ of 0.25 μg/mL. The *A. fumigatus* population from Guiyang exhibited the highest GM MIC and MIC_50_ values, which were 0.142 μg/mL and 0.125 μg/mL, respectively, while the highest MIC_90_ value (0.5 μg/mL) was observed in the Liupanshui population (Table 1). For voriconazole, the MIC distribution spanned 0.031 to 0.25 μg/mL, yielding a GM-MIC of 0.086 μg/mL, an MIC_50_ of 0.063 μg/mL, and an MIC_90_ of 0.125 μg/mL. The *A. fumigatus* population from Qianxinan exhibited the highest GM MIC and MIC_90_ values, which were 0.11 μg/mL and 0.25 μg/mL, respectively. The MIC_50_ values for Zunyi, Qiandongnan, Qianxinan, Liupanshui, and Tongren were all 0.125 μg/mL (Table 1). Interestingly, statistical analysis results indicated that there were significant differences in the MIC distributions of itraconazole and voriconazole among *A. fumigatus* from some geographical populations. For instance, in the itraconazole susceptibility test, the *A. fumigatus* population in Guiyang was significantly different from all other geographical populations except that in Liupanshui, while the Liupanshui population showed significant differences from both the Qianxinan and Qiandongnan populations. In the voriconazole susceptibility test, the *A. fumigatus* population in Guiyang exhibited significant differences from those in Zunyi, Tongren, Liupanshui, Qianxinan, and Qiandongnan (Figure 2).

### 3.2. cyp51A Polymorphism and Correlation of Mutation Sites with Triazole MICs

**Figure 3:**
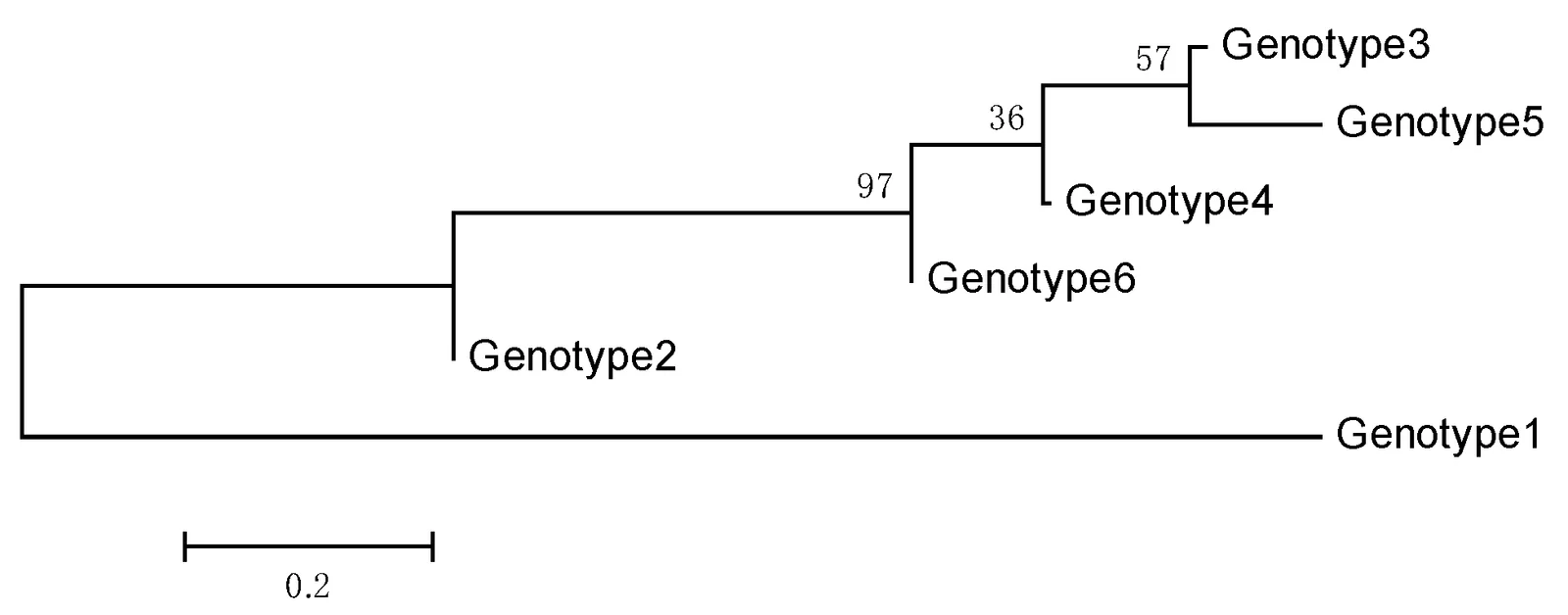
Phylogenetic relationships among the six *cyp51A* genotypes identified in this study. Branch lengths are proportional to nucleotide differences among the genotypes. Numbers along branches are bootstrap support values. Scale bar represents percentage of nucleotide sequence difference.

In this study, we successfully obtained the complete gene sequence of the *cyp51A* gene from 191 *A. fumigatus* strains. The GenBank accession numbers for the 191 cyp51A sequences of the 191 strains are PZ748371 to PZ748561. A total of 10 polymorphic sites (S) were detected and 6 haplotypes (h) were identified. The overall haplotype diversity (Hd) was 0.268, and the nucleotide diversity (Pi) was 0.00029 (Table 2). Sequence alignment comparisons with the reference sequence identified a total of 29 *A. fumigatus* strains carrying base substitutions in the *cyp51A* gene. The number of mutant strains and corresponding mutation frequencies in each geographic region were as follows: Bijie (6 strains, 28.57%, 6/21), Zunyi (2 strains, 9.52%, 2/21), Qiannan (2 strains, 9.52%, 2/21), Anshun (2 strains, 9.52%, 2/21), Tongren (3 strains, 14.29%, 3/21), Guiyang (3 strains, 13.64%, 3/22), Liupanshui (4 strains, 19.05%, 4/21), Qianxinan (4 strains, 19.05%, 4/21), and Qiandongnan (3 strains, 13.64%, 3/22). Among the 10 mutation sites located in exons, 4 were synonymous mutations (267G→A, 540G→A, 1074A→G, and 1362T→C), and 6 were non-synonymous mutations (137T→A, 514A→G, 743A→C, 744T→A, 765C→G, 1279G→A). The mutation at position 744 on the coding sequence had the highest frequency of 0.1204 (22/191), followed by positions 514 and 540, both with a frequency of 0.0209 (4/191) (Table S1). No mutations were found in the *cyp51A* gene of the remaining 162 *A. fumigatus* strains. Based on 10 polymorphic loci, the *cyp51A* gene of 191 *A. fumigatus* strains was classified into 6 genotypes. Genotype 6, which harbored wild-type strains, had the highest frequency at 0.839 (162/199). Genotype 5 was distributed across all 9 sampling sites. Genotype 1 was detected in Guiyang and Tongren, while Genotype 4 was found in Bijie and Qianxinan. Genotype 2 and Genotype 3 were identified exclusively in Bijie and Qiandongnan, respectively (Table 3). Phylogenetic analysis based on the *cyp51A* genotypes revealed that Genotype 1 exhibited the greatest genetic divergence from the other five genotypes. This genotype harbored eight specific variant loci at positions 137, 267, 514, 743, 765, 1074, 1279, and 1362. Genotype 4 and Genotype 6 were closely genetically related, with only a single nucleotide difference detected at position 540. The relationships among the six *cyp51A* genotypes are shown in Figure 3. Statistical analysis demonstrated that none of the 10 variant sites of the *cyp51A* gene (137, 267, 514, 540, 743, 744, 765, 1074, 1279, 1362) in this study showed any significant correlation with the minimum inhibitory concentration (MIC) values of itraconazole and voriconazole (*p* > 0.05) (Table 4).

### 3.3. cyp51A Amino Acid Substitution and Phylogenetic Analysis

Of the 29 *A. fumigatus* strains harboring *cyp51A* gene mutations, 25 exhibited amino acid substitutions at the protein level, including 4 strains from Bijie (out of 6 strains with nucleotide substitutions), 4 from Liupanshui, 3 from Tongren, Guiyang, and Qiandongnan each, and 2 from Zunyi, Qiannan, Anshun, and Qianxinan (out of 4 strains with nucleotide substitutions) each. A total of 10 variant loci in the coding sequences resulted in amino acid substitutions at 5 positions of the protein encoded by the *cyp51A* gene, namely F46Y, M172V, N248T/K, D255E, and E427K. Among these substitutions, N248T/K at position 248 exhibited the highest frequency and was distributed across all sampling sites, showing obvious geographical broad-spectrum characteristics, with an occurrence frequency of 0.1309 (25/191). The remaining four substitution loci (F46Y, M172V, D255E, and E427K) all had a frequency of 0.0105 (2/191) and were only detected in the Guiyang and Tongren sampling sites (Table 5).

Phylogenetic analysis was performed using 1494 *A. fumigatus cyp51A* sequences, including 1303 sequences downloaded from the NCBI database and 191 sequences obtained from isolates collected in Guizhou. The resulting phylogenetic tree was divided into two clusters (A and B). Cluster A comprised 1484 sequences with wide geographical representation, whereas Clade B contained 10 sequences originating from the USA (9 sequences) and Spain (1 sequence). All the six sequence types from our current study belonged to cluster A. While there were some geographically specific subclusters within cluster A, the six sequence types from this study were broadly distributed across the cluster A portion of the *cyp51A* gene tree (Figure S1).

## 4. Discussion

### 4.1. Absence of Triazole-Resistant A. fumigatus Strains

In this study, we systematically conducted azole antifungal susceptibility testing on 191 strains of *A. fumigatus* isolated from the soil of 9 tea plantations in Guizhou Province, the core green tea-producing region of China. Among them, the susceptibility test results for itraconazole and voriconazole showed that the MIC of all tested strains was below 1.0 µg/mL, and no azole-resistant strain was detected. This indicates that *A. fumigatus* isolates in the soil of Guizhou tea plantations are highly sensitive to the above two azole drugs. In an earlier study, we conducted a similar investigation on ARAF using the soil of 9 rural vegetable gardens across Guizhou Province as the research object. The results showed that the incidence of azole resistance was only 0.49% (only 1 out of 206 strains was resistant) [4]. The findings of these two studies suggest that the prevalence of ARAF in agricultural soil samples from Guizhou Province is extremely low, much lower than the findings of previous investigations conducted in the neighboring Yunnan Province [17,32,33] and other regions of China [39,40]. Globally, although a similarly low triazole resistance frequency of 0.27% has been reported in soil *A. fumigatus* populations in Canada [41], azole resistance rates in *A. fumigatus* from most regions worldwide are generally much higher [42]. The resistance rate reaches up to 50% in some areas outside China [43,44,45] and reaches nearly 80% in greenhouses around Kunming, Yunnan Province, southwest China [17,40].

In the previous study on *A. fumigatus* from vegetable garden soils in Guizhou, we speculated that the low incidence of ARAF in Guizhou Province might be related to four factors [4]. The first was strict control of agricultural pesticide use by local governments, which effectively restricts the abusive use of azole fungicides. The second was the long-term adherence of local farmers to traditional green agriculture practices, which reduce their dependence on chemical pesticides. The third was the unique karst landforms and the perennial low-temperature and humid climatic characteristics of Guizhou Province, which may have a certain inhibitory effect on the growth and reproduction of *A. fumigatus* and its azole resistance mutations. The fourth was the limited gene flow of *A. fumigatus* among different geographical populations, making it difficult to achieve the broad distribution of azole resistance genes [4]. The sampling sites of soil samples collected from tea-growing areas in this study were highly consistent with those of previous vegetable garden soil samples, both located in typical agricultural planting areas in Guizhou Province. Therefore, it is speculated that the core reasons for the low incidence of ARAF are consistent with those in the previous study. In addition, through in-depth communication with local tea planting experts, it was learned that the overall incidence of fungal diseases in Guizhou tea planting is relatively low; and for occasional tea fungal diseases, local farmers generally adopt the prevention and control measure of “directly destroying diseased plants in the early stage of infection”. This measure can eliminate the spread and transmission of fungal pathogens from the source, thereby reducing the usage of various chemical pesticides, including triazole fungicides, further reducing the probability of *A. fumigatus* being exposed to azole fungicides, and providing additional guarantee for the maintenance of its low azole resistance level.

### 4.2. Non-Azole-Resistance Mutation Sites in the cyp51A Gene

In the present study, the *cyp51A* genes of 191 environmental *A. fumigatus* isolates were sequenced and analyzed. A total of 29 isolates were identified to harbor different base substitution mutations, with an overall mutation frequency of 15.19% (29/191). Combined with the in vitro antifungal susceptibility data, further analysis showed that these *cyp51A* base substitutions did not lead to a notable increase in the MIC of itraconazole and voriconazole, and no significant changes were observed in the drug-sensitive phenotypes of the isolates. This finding is consistent with the molecular epidemiological results of *A. fumigatus* reported worldwide [28,35,42,46,47,48], suggesting that abundant non-resistance-related genetic variations commonly exist in the *cyp51A* gene of natural *A. fumigatus* populations. Most gene mutations without phenotypic differences in drug susceptibility are silent or neutral variations, which only reflect genetic polymorphisms at the nucleotide level and cannot directly mediate the emergence of triazole-resistant phenotypes [28,47]. At present, the evolutionary pressure, environmental selection factors and molecular regulatory mechanisms responsible for the high prevalence of non-resistant mutations in the *cyp51A* gene remain unclear. Further investigations with expanded sample sizes, combined with regional environmental features and molecular evolutionary analysis, are therefore needed to systematically clarify the underlying causes and biological significance of these mutations.

### 4.3. Widespread Distribution of CYP51A Mutations

In this study, five amino acid substitution mutations were identified in the *cyp51A* gene, including F46Y, M172V, N248T/K, D255E, and E427K. Among all mutation variants, N248K presented the highest overall detection frequency with a mutation rate of 0.1205 (23/191). This mutation was widely distributed across all nine geographical sampling areas surveyed in this study, and its spatial distribution pattern was highly consistent with previous investigations of natural *A. fumigatus* populations across different regions of China [2,17,42,49,50]. Accumulating clinical studies have demonstrated that the N248K variation can reduce the susceptibility of *A. fumigatus* to azole antifungal agents and weaken their inhibitory activity, thereby exerting adverse impacts on the clinical management of fungal infections [48,51]. Nevertheless, numerous epidemiological investigations and molecular phenotypic analyses have further confirmed that environmental *A. fumigatus* strains carrying only the single N248K mutation remain phenotypically sensitive to conventional azoles. This single-site variation cannot independently mediate the development of drug resistance and shows no significant statistical correlation with the resistant phenotype [28,49,50]. The above research conclusions are completely consistent with the results of drug susceptibility testing and genetic variation analysis obtained in the present study.

In this study, a total of two *A. fumigatus* isolates carrying five amino acid substitution mutation sites, namely F46Y, M172V, N248T, D255E and E427K, were identified from the populations in Tongren and Guiyang. These five mutations occurred in linkage. The distribution pattern of this compound mutation is consistent with previous studies on *A. fumigatus* populations in other regions of China [17,49,52] and across the globe [28,47,53], which further confirms the prevalence of such multi-site combined mutations in environmental strains. Antifungal susceptibility tests showed that the two above-mentioned isolates, derived from Tongren and Guiyang respectively, had itraconazole minimum inhibitory concentration (MIC) values of 0.125 µg/mL and 0.25 µg/mL, and both exhibited a voriconazole MIC of 0.063 µg/mL. All MIC values were lower than the clinical resistance breakpoints for azole drugs, and the isolates remained phenotypically sensitive with no azole resistance observed. The results indicate that this multi-site compound mutation pattern cannot induce azole resistance in *A. fumigatus*, which is highly consistent with the findings of existing domestic [49,50] and international studies [28,54,55].

Of the six *cyp51A* sequence types identified here, three (genotypes 2, 3, and 4) were newly reported in this study and found so far only in Guizhou. Two of these three genotypes (genotypes 2 and 3) were singletons, represented by only one strain each, while genotype 4 was represented by three strains. This result is consistent with at least some unique genetic diversity of *cyp51A* in *A. fumigatus* in Guizhou. In contrast, the more common genotypes in Guizhou, genotypes 1, 5, and 6, were shared broadly with strains from other regions in China as well as from outside of China, consistent with the Guizhou population of *A. fumigatus* being part of the global metapopulation. Interestingly, *cyp51A* genotypes 1, 5, and 6 were not clustered together on the phylogenetic tree but were broadly distributed, indicating recent or ongoing genetic exchanges among geographic populations of *A. fumigatus* between Guizhou and those from outside of Guizhou.

## 5. Conclusions

This study isolated and analyzed 191 *A. fumigatus* strains from the soils of nine tea plantations across nine regions in Guizhou Province in southwestern China. Antifungal susceptibility tests indicated that all isolates were sensitive to two commonly used triazole antifungals, with MIC values no more than 0.5 μg/mL. Non-resistance-related mutations in the *cyp51A* gene were detected in 29 strains, yielding a mutation frequency of 15.18% (29/191). Notably, the nucleotide mutation at position 744 in the coding region of *cyp51A* was prevalent among all sampling sites, with a frequency of 12.04% (23/191). This study demonstrates that environmental *A. fumigatus* strains harbor abundant naturally occurring genetic variations that are not associated with triazole resistance. Our results further support that traditional farming with limited agricultural fungicide usage does not facilitate the emergence and spread of drug-resistant fungal pathogens. Whenever possible, such a practice should be preserved and promoted for better human health [56].

## Figures and Tables

**Table 1:** Analysis results of susceptibility testing of *A. fumigatus* to Itraconazole and Voriconazole. GM refers to geometric mean. MIC_50_ refers to the lowest concentration able to inhibit the growth of 50% of the strains. MIC_90_ represents the lowest concentration that inhibits the growth of 90% of the strains.

Geographical Source	No. of Strains	MIC (µg/mL)							
		Itraconazole				Voriconazole			
		Range	GM	MIC_50_	MIC_90_	Range	GM	MIC_50_	MIC_90_
Guiyang	22	0.031–0.5	0.142	0.125	0.25	0.031–0.125	0.053	0.063	0.063
Zunyi	21	0.031–0.25	0.074	0.063	0.125	0.063–0.125	0.099	0.125	0.125
Qiannan	21	0.031–0.125	0.05	0.063	0.125	0.063–0.125	0.079	0.063	0.125
Anshun	21	0.031–0.125	0.067	0.063	0.125	0.031–0.125	0.076	0.063	0.125
Qiandongnan	22	0.031–0.125	0.055	0.063	0.125	0.063–0.125	0.104	0.125	0.125
Qianxinan	21	0.015–0.25	0.039	0.031	0.25	0.063–0.25	0.11	0.125	0.25
Liupanshui	21	0.031–0.5	0.084	0.063	0.5	0.031–0.125	0.093	0.125	0.125
Bijie	21	0.031–0.125	0.069	0.063	0.125	0.031–0.25	0.082	0.063	0.125
Tongren	21	0.031–0.125	0.063	0.063	0.125	0.063–0.25	0.096	0.125	0.125
Total	191	0.015–0.5	0.067	0.063	0.25	0.031–0.25	0.086	0.063	0.125

**Table 2:** Analysis of nucleotide polymorphism in the *cyp51A* gene of *A. fumigatus* from different geographical populations.

Geographical Source	No. of Strains	Number of Polymorphic Sites (S)	Number of Haplotypes (h)	Haplotype Diversity (Hd)	Nucleotide Diversity (Pi)
Guiyang	22	9	3	0.255	0.00058
Zunyi	21	1	2	0.181	0.00012
Qiannan	21	1	2	0.181	0.00012
Anshun	21	1	2	0.181	0.00012
Qiandongnan	22	2	3	0.255	0.00022
Qianxinan	21	2	3	0.343	0.00023
Liupanshui	21	1	2	0.324	0.00021
Bijie	21	5	4	0.471	0.00046
Tongren	21	9	3	0.267	0.00061
Total	191	10	6	0.268	0.00029

**Table 3:** Information of representative *cyp51A* gene sequence types of *A. fumigatus* strains from different geographical origins.

Geographical Source	No. of Strains	No. of Genotypes	No. of Isolates for Each Genotype					
			1	2	3	4	5	6
Guiyang	22	3	1				2	19
Zunyi	21	2					2	19
Qiannan	21	2					2	19
Anshun	21	2					2	19
Qiandongnan	22	3			1		2	19
Qianxinan	21	3				2	2	17
Liupanshui	21	2					4	17
Bijie	21	4		1		1	4	15
Tongren	21	3	1				2	18
Total	191	6	2	1	1	3	22	162

**Table 4:** Correlation Analysis of *cyp51A* Gene Variant Loci with Itraconazole and Voriconazole MIC Values in *A. fumigatus*.

*cyp51A* Gene Mutation Sites	Itraconazole		Voriconazole	
	Correlation Coefficient	*p*-Value	Correlation Coefficient	*p*-Value
137	−0.1365	0.0597	0.0872	0.2305
267	−0.0605	0.4058	−0.0081	0.9118
514	0.1365	0.0597	−0.0872	0.2305
540	0.0699	0.3365	−0.0686	0.3456
743	0.1365	0.0597	−0.0872	0.2305
744	−0.0436	0.5491	−0.0101	0.8901
765	0.1365	0.0597	−0.0872	0.2305
1074	0.0605	0.4058	0.0081	0.9118
1279	−0.1365	0.0597	0.0872	0.2305
1362	−0.0605	0.4058	−0.0081	0.9118

**Table 5:** Antifungal susceptibility and amino acid substitutions in CYP51A of 29 *A. fumigatus* isolates. MIC: minimum inhibitory concentration; ITR: itraconazole; VOR: voriconazole.

Geographical Source	Strain Number	MIC (µg/mL)		CYP51A Substitutions (*cyp51A* Gene Mutation Sites)
		ITR	VOR	
Bijie	BJ-02	0.031	0.25	Synonymous substitutions (267, 540, 1362)
	BJ-11	0.125	0.063	N248K (744)
	BJ-12	0.125	0.063	Synonymous substitution (540)
	BJ-13	0.125	0.063	N248K (744)
	BJ-15	0.063	0.125	N248K (744)
	BJ-20	0.125	0.063	N248K (744)
Zunyi	ZY-06	0.063	0.063	N248K (744)
	ZY-19	0.125	0.125	N248K (744)
Qiannan	QN-02	0.031	0.063	N248K (744)
	QN-08	0.031	0.125	N248K (744)
Anshun	AS-02	0.063	0.063	N248K (744)
	AS-17	0.063	0.125	N248K (744)
Tongren	TR-06	0.063	0.125	N248K (744)
	TR-12	0.125	0.063	N248K (744)
	TR-21	0.125	0.063	F46Y, M172V, N248T, D255E, E427K (137, 267, 514, 743, 765, 1074, 1279, 1362)
Guiyang	GY-01	0.063	0.063	N248K (744)
	GY-08	0.25	0.063	F46Y, M172V, N248T, D255E, E427K (137, 267, 514, 743, 765, 1074, 1279, 1362)
	GY-14	0.25	0.031	N248K (744)
Liupanshui	LPS-06	0.125	0.125	N248K (744)
	LPS-07	0.063	0.063	N248K (744)
	LPS-08	0.063	0.125	N248K (744)
	LPS-19	0.031	0.125	N248K (744)
Qianxinnan	QXN-01	0.25	0.125	N248K (744)
	QXN-09	0.015	0.063	N248K (744)
	QXN-13	0.015	0.125	Synonymous substitution (540)
	QXN-17	0.031	0.125	Synonymous substitution (540)
Qiandongnan	QDN-01	0.063	0.125	N248K (744)
	QDN-02	0.031	0.125	N248K (744)
	QDN-08	0.063	0.125	N248K (540, 744)

## Data Availability

The original contributions presented in this study are included in the article/Supplementary Material. Further inquiries can be directed to the corresponding authors. DNA sequences at the *cyp51A* gene for the 191 strains of *Aspergillus fumigatus* have been deposited into GenBank under accession numbers PZ748371 to PZ748561.

## Supplementary Materials

The following supporting information can be downloaded at: https://www.mdpi.com/article/10.3390/microorganisms14081704/s1, Table S1: Information on variant sites of the *cyp51A* gene coding sequence in *A. fumigatus*; Figure S1. Global phylogenetic analysis of *A. fumigatus cyp51A* from different geographic sources.

## Abbreviations

The following abbreviations are used in this manuscript:

ARAF	azole-resistant *A. fumigatus*
ITR	itraconazole
VOR	voriconazole
MIC	minimum inhibitory concentration
