# Supplementary material for: Antifungal Susceptibility and Cyp51A Gene Variation Analysis of Aspergillus fumigatus Isolated from Soils in Tea-Growing Areas of Guizhou, China
Source: Microorganisms. 2026 Aug 4;14(8):1704. doi: 10.3390/microorganisms14081704 (PMC13515310; doi:10.3390/microorganisms14081704)
Supplement: Supplementary file 1 [file microorganisms-14-01704-s001.zip › Figure S1. Global phylogenetic analysis of A. fumigatus cyp51A from different geographic sources.pdf]

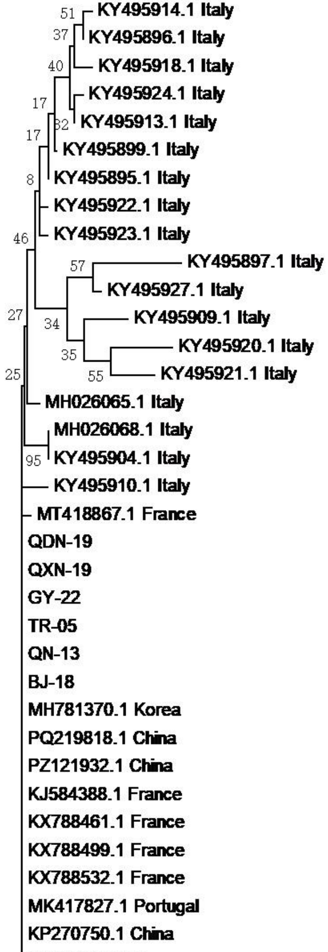

KY495914.1 Italy
KY495896.1 Italy
KY495918.1 Italy
KY495924.1 Italy
KY495913.1 Italy
KY495899.1 Italy
KY495895.1 Italy
KY495922.1 Italy
KY495923.1 Italy
KY495897.1 Italy
KY495927.1 Italy
KY495909.1 Italy
KY495920.1 Italy
KY495921.1 Italy
MH026065.1 Italy
MH026068.1 Italy
KY495904.1 Italy
KY495910.1 Italy
MT418867.1 France
QDN-19
QXN-19
GY-22
TR-05
QN-13
BJ-18
MH781370.1 Korea
PQ219818.1 China
PZ121932.1 China
KJ584388.1 France
KX788461.1 France
KX788499.1 France
KX788532.1 France
MK417827.1 Portugal
KP270750.1 China
51
37
40
17
32
17
8
46
57
27
34
35
25
55
95

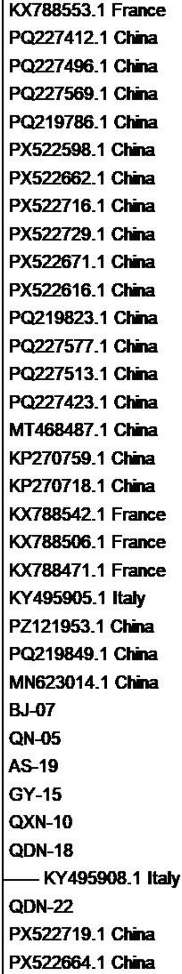
KX788553.1 France
PQ227412.1 China
PQ227496.1 China
PQ227569.1 China
PQ219786.1 China
PX522598.1 China
PX522662.1 China
PX522716.1 China
PX522729.1 China
PX522671.1 China
PX522616.1 China
PQ219823.1 China
PQ227577.1 China
PQ227513.1 China
PQ227423.1 China
MT468487.1 China
KP270759.1 China
KP270718.1 China
KX788542.1 France
KX788506.1 France
KX788471.1 France
KY495905.1 Italy
PZ121953.1 China
PQ219849.1 China
MN623014.1 China
BJ-07
QN-05
AS-19
GY-15
QXN-10
QDN-18
KY495908.1 Italy
QDN-22
PX522719.1 China
PX522664.1 China

PX522599.1 China
PQ219788.1 China
PQ227570.1 China
PQ227497.1 China
PQ227415.1 China
KX788554.1 France
KP270751.1 China
MH026075.1 Italy
KX788533.1 France
KX788500.1 France
KX788462.1 France
KJ584391.1 France
PZ121934.1 China
PQ219820.1 China
MH781373.1 Korea
BJ-17
QN-12
TR-04
GY-21
MK417817.1 Portugal
QXN-18
PX522748.1 China
PX522686.1 China
PX522640.1 China
PQ219854.1 China
PQ227604.1 China
PQ227532.1 China
PQ227448.1 China
MT309495.1 France
MT468488.1 China
KP270732.1 China
KX788562.1 France
KX788518.1 France
KX788484.1 France

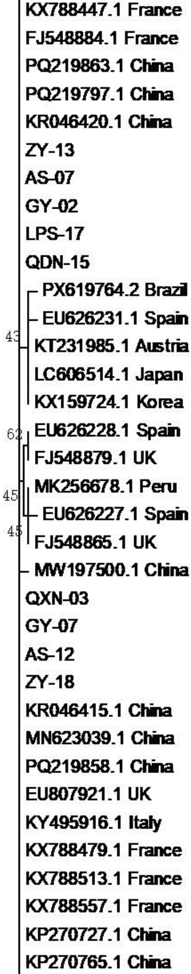
KX788447.1 France
FJ548884.1 France
PQ219863.1 China
PQ219797.1 China
KR046420.1 China
ZY-13
AS-07
GY-02
LPS-17
QDN-15
PX619764.2 Brazil
EU626231.1 Spain
43
KT231985.1 Austria
LC606514.1 Japan
KX159724.1 Korea
62
EU626228.1 Spain
FJ548879.1 UK
MK256678.1 Peru
45
EU626227.1 Spain
45
FJ548865.1 UK
MW197500.1 China
QXN-03
GY-07
AS-12
ZY-18
KR046415.1 China
MN623039.1 China
PQ219858.1 China
EU807921.1 UK
KY495916.1 Italy
KX788479.1 France
KX788513.1 France
KX788557.1 France
KP270727.1 China
KP270765.1 China

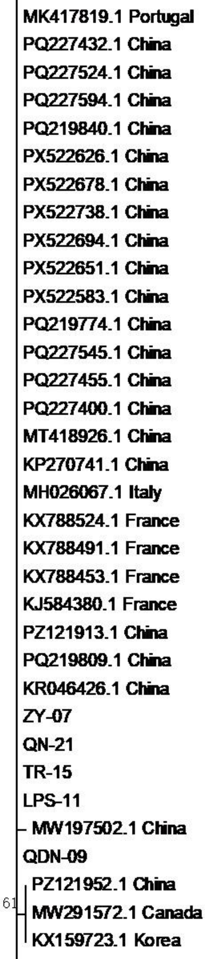
MK417819.1 Portugal
PQ227432.1 China
PQ227524.1 China
PQ227594.1 China
PQ219840.1 China
PX522626.1 China
PX522678.1 China
PX522738.1 China
PX522694.1 China
PX522651.1 China
PX522583.1 China
PQ219774.1 China
PQ227545.1 China
PQ227455.1 China
PQ227400.1 China
MT418926.1 China
KP270741.1 China
MH026067.1 Italy
KX788524.1 France
KX788491.1 France
KX788453.1 France
KJ584380.1 France
PZ121913.1 China
PQ219809.1 China
KR046426.1 China
ZY-07
QN-21
TR-15
LPS-11
MW197502.1 China
QDN-09
PZ121952.1 China
61
MW291572.1 Canada
KX159723.1 Korea

QXN-16
GY-20
TR-03
QN-11
BJ-16
MH781377.1 Korea
PQ219822.1 China
PZ121935.1 China
KY495898.1 Italy
KX788466.1 France
KX788501.1 France
KX788536.1 France
MH026076.1 Italy
KP270753.1 China
KX788555.1 France
PQ227416.1 China
PQ227498.1 China
PQ227572.1 China
PQ219789.1 China
PX522602.1 China
PX522665.1 China
PX522722.1 China
PQ219846.1 China
QXN-06
GY-11
AS-15
QN-01
BJ-04
MN623036.1 China
PQ219853.1 China
PZ121962.1 China
KY495911.1 Italy
KX788474.1 France
KX788510.1 France
KX788545.1 France

KP270723.1 China
KP270762.1 China
MH231597.1 Portugal
PQ227427.1 China
PQ227520.1 China
PQ227584.1 China
PQ219832.1 China
PX522622.1 China
PX522675.1 China
PX522735.1 China
PX522690.1 China
PX522645.1 China
PX522572.1 China
PQ227617.1 China
PQ227541.1 China
PQ227452.1 China
PQ227395.1 China
MT418920.1 China
KP270736.1 China
MH026063.1 Italy
KX788521.1 France
KX788487.1 France
KX788450.1 France
KJ584377.1 France
PZ121908.1 China
PQ219805.1 China
KR046423.1 China
ZY-10
AS-04
TR-18
LPS-14
MW197499.1 China
QDN-12

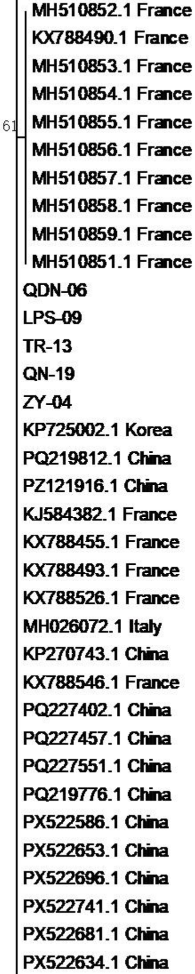
MH510852.1 France
KX788490.1 France
MH510853.1 France
MH510854.1 France
61
MH510855.1 France
MH510856.1 France
MH510857.1 France
MH510858.1 France
MH510859.1 France
MH510851.1 France
QDN-06
LPS-09
TR-13
QN-19
ZY-04
KP725002.1 Korea
PQ219812.1 China
PZ121916.1 China
KJ584382.1 France
KX788455.1 France
KX788493.1 France
KX788526.1 France
MH026072.1 Italy
KP270743.1 China
KX788546.1 France
PQ227402.1 China
PQ227457.1 China
PQ227551.1 China
PQ219776.1 China
PX522586.1 China
PX522653.1 China
PX522696.1 China
PX522741.1 China
PX522681.1 China
PX522634.1 China

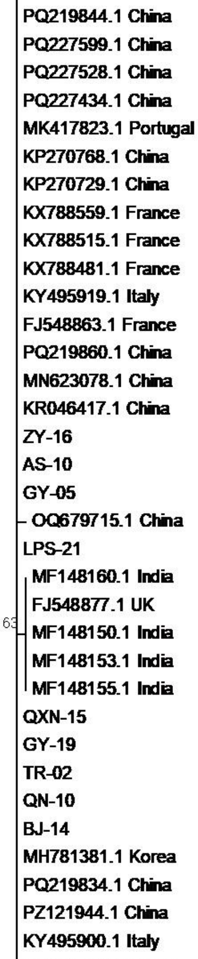
PQ219844.1 China
PQ227599.1 China
PQ227528.1 China
PQ227434.1 China
MK417823.1 Portugal
KP270768.1 China
KP270729.1 China
KX788559.1 France
KX788515.1 France
KX788481.1 France
KY495919.1 Italy
FJ548863.1 France
PQ219860.1 China
MN623078.1 China
KR046417.1 China
ZY-16
AS-10
GY-05
OQ679715.1 China
LPS-21
MF148160.1 India
FJ548877.1 UK
63
MF148150.1 India
MF148153.1 India
MF148155.1 India
QXN-15
GY-19
TR-02
QN-10
BJ-14
MH781381.1 Korea
PQ219834.1 China
PZ121944.1 China
KY495900.1 Italy

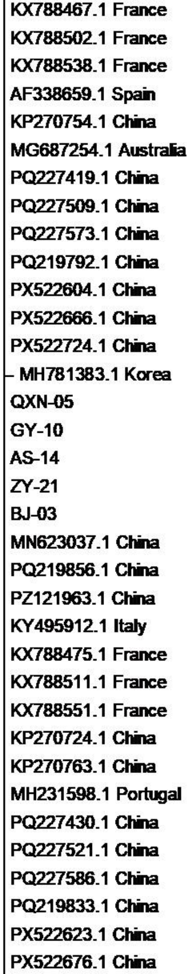
KX788467.1 France
KX788502.1 France
KX788538.1 France
AF338659.1 Spain
KP270754.1 China
MG687254.1 Australia
PQ227419.1 China
PQ227509.1 China
PQ227573.1 China
PQ219792.1 China
PX522604.1 China
PX522666.1 China
PX522724.1 China
MH781383.1 Korea
QXN-05
GY-10
AS-14
ZY-21
BJ-03
MN623037.1 China
PQ219856.1 China
PZ121963.1 China
KY495912.1 Italy
KX788475.1 France
KX788511.1 France
KX788551.1 France
KP270724.1 China
KP270763.1 China
MH231598.1 Portugal
PQ227430.1 China
PQ227521.1 China
PQ227586.1 China
PQ219833.1 China
PX522623.1 China
PX522676.1 China

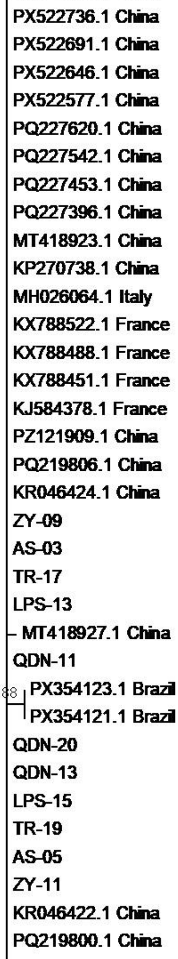

PX522736.1 China
PX522691.1 China
PX522646.1 China
PX522577.1 China
PQ227620.1 China
PQ227542.1 China
PQ227453.1 China
PQ227396.1 China
MT418923.1 China
KP270738.1 China
MH026064.1 Italy
KX788522.1 France
KX788488.1 France
KX788451.1 France
KJ584378.1 France
PZ121909.1 China
PQ219806.1 China
KR046424.1 China
ZY-09
AS-03
TR-17
LPS-13
MT418927.1 China
QDN-11
88
PX354123.1 Brazil
PX354121.1 Brazil
QDN-20
QDN-13
LPS-15
TR-19
AS-05
ZY-11
KR046422.1 China
PQ219800.1 China

PX619774.2 China
KJ584376.1 France
KX788449.1 France
KX788486.1 France
KX788520.1 France
MH026062.1 Italy
KP270735.1 China
MT418918.1 China
PQ227394.1 China
PQ227450.1 China
PQ227535.1 China
PQ227614.1 China
PX522571.1 China
PX522643.1 China
PX522689.1 China
PX522734.1 China
PX522674.1 China
PX522621.1 China
PQ219829.1 China
PQ227581.1 China
PQ227517.1 China
PQ227426.1 China
MH231596.1 Portugal
KP270761.1 China
KP270722.1 China
KX788544.1 France
KX788508.1 France
KX788473.1 France
KY495907.1 Italy
PZ121961.1 China
PQ219852.1 China
MN623034.1 China
BJ-05
QN-03
AS-16

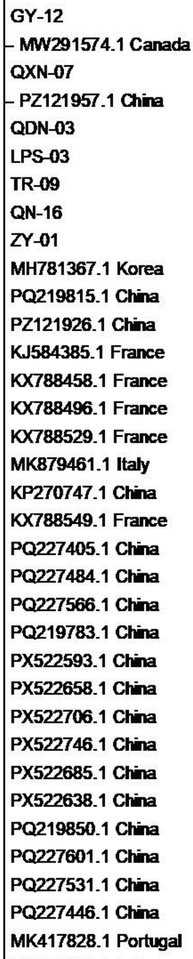
GY-12
MW291574.1 Canada
QXN-07
PZ121957.1 China
QDN-03
LPS-03
TR-09
QN-16
ZY-01
MH781367.1 Korea
PQ219815.1 China
PZ121926.1 China
KJ584385.1 France
KX788458.1 France
KX788496.1 France
KX788529.1 France
MK879461.1 Italy
KP270747.1 China
KX788549.1 France
PQ227405.1 China
PQ227484.1 China
PQ227566.1 China
PQ219783.1 China
PX522593.1 China
PX522658.1 China
PX522706.1 China
PX522746.1 China
PX522685.1 China
PX522638.1 China
PQ219850.1 China
PQ227601.1 China
PQ227531.1 China
PQ227446.1 China
MK417828.1 Portugal

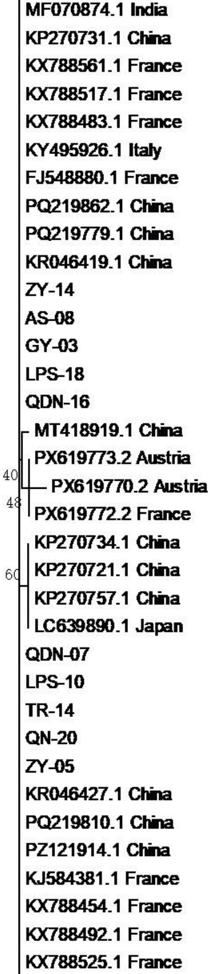

MF070874.1 India
KP270731.1 China
KX788561.1 France
KX788517.1 France
KX788483.1 France
KY495926.1 Italy
FJ548880.1 France
PQ219862.1 China
PQ219779.1 China
KR046419.1 China
ZY-14
AS-08
GY-03
LPS-18
QDN-16
MT418919.1 China
PX619773.2 Austria
40
PX619770.2 Austria
48
PX619772.2 France
KP270734.1 China
KP270721.1 China
60
KP270757.1 China
LC639890.1 Japan
QDN-07
LPS-10
TR-14
QN-20
ZY-05
KR046427.1 China
PQ219810.1 China
PZ121914.1 China
KJ584381.1 France
KX788454.1 France
KX788492.1 France
KX788525.1 France

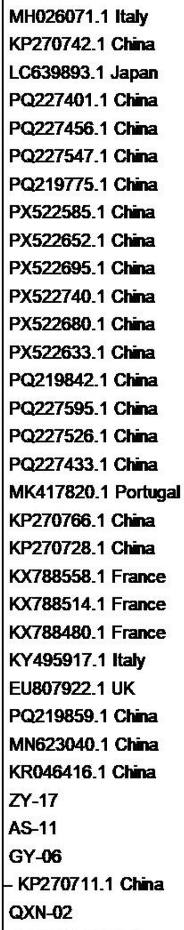
MH026071.1 Italy
KP270742.1 China
LC639893.1 Japan
PQ227401.1 China
PQ227456.1 China
PQ227547.1 China
PQ219775.1 China
PX522585.1 China
PX522652.1 China
PX522695.1 China
PX522740.1 China
PX522680.1 China
PX522633.1 China
PQ219842.1 China
PQ227595.1 China
PQ227526.1 China
PQ227433.1 China
MK417820.1 Portugal
KP270766.1 China
KP270728.1 China
KX788558.1 France
KX788514.1 France
KX788480.1 France
KY495917.1 Italy
EU807922.1 UK
PQ219859.1 China
MN623040.1 China
KR046416.1 China
ZY-17
AS-11
GY-06
KP270711.1 China
QXN-02

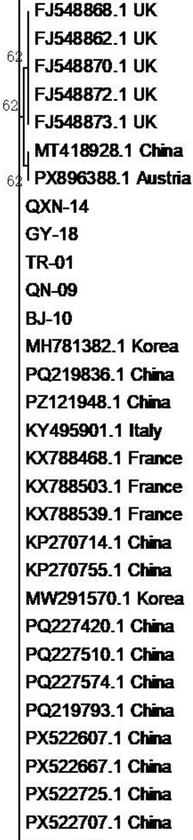
FJ548868.1 UK
FJ548862.1 UK
62
FJ548870.1 UK
FJ548872.1 UK
62
FJ548873.1 UK
MT418928.1 China
62
PX896388.1 Austria
QXN-14
GY-18
TR-01
QN-09
BJ-10
MH781382.1 Korea
PQ219836.1 China
PZ121948.1 China
KY495901.1 Italy
KX788468.1 France
KX788503.1 France
KX788539.1 France
KP270714.1 China
KP270755.1 China
MW291570.1 Korea
PQ227420.1 China
PQ227510.1 China
PQ227574.1 China
PQ219793.1 China
PX522607.1 China
PX522667.1 China
PX522725.1 China
PX522707.1 China

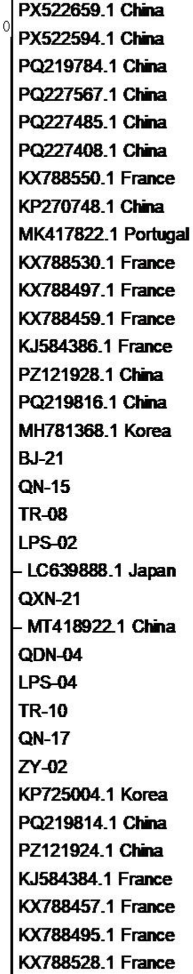

0
PX522659.1 China
PX522594.1 China
PQ219784.1 China
PQ227567.1 China
PQ227485.1 China
PQ227408.1 China
KX788550.1 France
KP270748.1 China
MK417822.1 Portugal
KX788530.1 France
KX788497.1 France
KX788459.1 France
KJ584386.1 France
PZ121928.1 China
PQ219816.1 China
MH781368.1 Korea
BJ-21
QN-15
TR-08
LPS-02
LC639888.1 Japan
QXN-21
MT418922.1 China
QDN-04
LPS-04
TR-10
QN-17
ZY-02
KP725004.1 Korea
PQ219814.1 China
PZ121924.1 China
KJ584384.1 France
KX788457.1 France
KX788495.1 France
KX788528.1 France

MK879460.1 Italy
KP270745.1 China
KX788548.1 France
PQ227404.1 China
PQ227460.1 China
PQ227559.1 China
PQ219781.1 China
PX522592.1 China
PX522657.1 China
PX522705.1 China
PX522573.1 China
MW197503.1 China
QXN-08
GY-13
AS-18
QN-04
BJ-06
MN623031.1 China
PQ219851.1 China
PZ121960.1 China
KY495906.1 Italy
KX788472.1 France
KX788507.1 France
KX788543.1 France
KP270720.1 China
KP270760.1 China
MH026070.1 Italy
PQ227425.1 China
PQ227515.1 China
PQ227580.1 China
PQ219825.1 China
PX522620.1 China
PX522673.1 China
PX522732.1 China

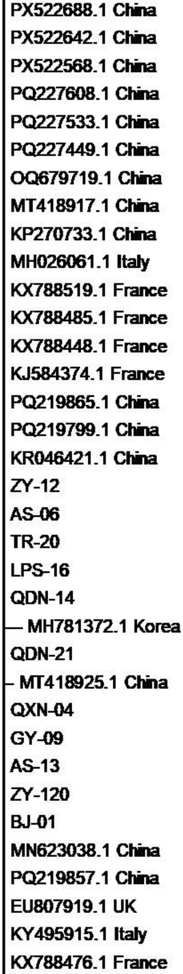
PX522688.1 China
PX522642.1 China
PX522568.1 China
PQ227608.1 China
PQ227533.1 China
PQ227449.1 China
OQ679719.1 China
MT418917.1 China
KP270733.1 China
MH026061.1 Italy
KX788519.1 France
KX788485.1 France
KX788448.1 France
KJ584374.1 France
PQ219865.1 China
PQ219799.1 China
KR046421.1 China
ZY-12
AS-06
TR-20
LPS-16
QDN-14
MH781372.1 Korea
QDN-21
MT418925.1 China
QXN-04
GY-09
AS-13
ZY-120
BJ-01
MN623038.1 China
PQ219857.1 China
EU807919.1 UK
KY495915.1 Italy
KX788476.1 France

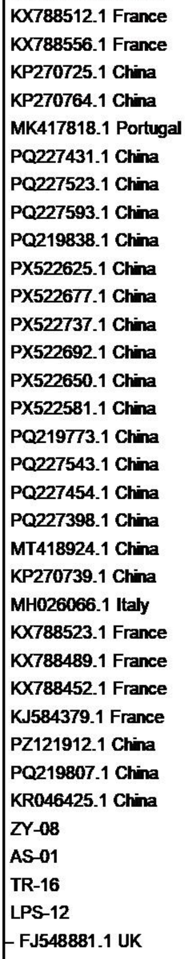
KX788512.1 France
KX788556.1 France
KP270725.1 China
KP270764.1 China
MK417818.1 Portugal
PQ227431.1 China
PQ227523.1 China
PQ227593.1 China
PQ219838.1 China
PX522625.1 China
PX522677.1 China
PX522737.1 China
PX522692.1 China
PX522650.1 China
PX522581.1 China
PQ219773.1 China
PQ227543.1 China
PQ227454.1 China
PQ227398.1 China
MT418924.1 China
KP270739.1 China
MH026066.1 Italy
KX788523.1 France
KX788489.1 France
KX788452.1 France
KJ584379.1 France
PZ121912.1 China
PQ219807.1 China
KR046425.1 China
ZY-08
AS-01
TR-16
LPS-12
FJ548881.1 UK

QDN-10
LC639889.1 Japan
QXN-20
LPS-01
TR-07
QN-14
BJ-19
MH781369.1 Korea
PQ219817.1 China
PZ121929.1 China
KJ584387.1 France
KX788460.1 France
KX788498.1 France
KX788531.1 France
MK417826.1 Portugal
KP270749.1 China
KX788552.1 France
PQ227410.1 China
PQ227491.1 China
PQ227568.1 China
PQ219785.1 China
PX522596.1 China
PX522661.1 China
PX522708.1 China
PX522726.1 China
PX522668.1 China
PX522609.1 China
PQ219796.1 China
PQ227575.1 China
PQ227511.1 China
PQ227421.1 China
MZ673600.1 Brazil
KP270756.1 China
KP270716.1 China
KX788540.1 France

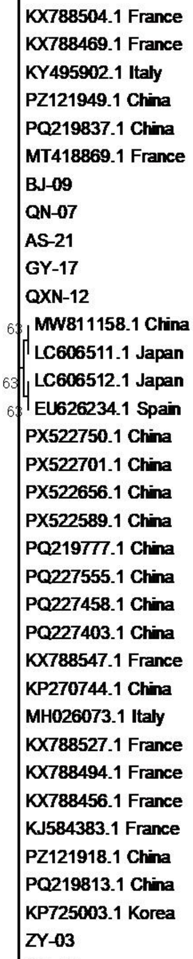
KX788504.1 France
KX788469.1 France
KY495902.1 Italy
PZ121949.1 China
PQ219837.1 China
MT418869.1 France
BJ-09
QN-07
AS-21
GY-17
QXN-12
63
MW811158.1 China
LC606511.1 Japan
63
LC606512.1 Japan
63
EU626234.1 Spain
PX522750.1 China
PX522701.1 China
PX522656.1 China
PX522589.1 China
PQ219777.1 China
PQ227555.1 China
PQ227458.1 China
PQ227403.1 China
KX788547.1 France
KP270744.1 China
MH026073.1 Italy
KX788527.1 France
KX788494.1 France
KX788456.1 France
KJ584383.1 France
PZ121918.1 China
PQ219813.1 China
KP725003.1 Korea
ZY-03

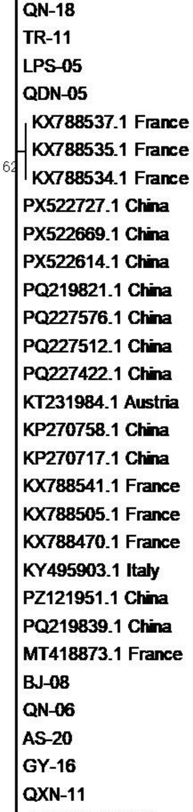

QN-18
TR-11
LPS-05
QDN-05
KX788537.1 France
KX788535.1 France
62
KX788534.1 France
PX522727.1 China
PX522669.1 China
PX522614.1 China
PQ219821.1 China
PQ227576.1 China
PQ227512.1 China
PQ227422.1 China
KT231984.1 Austria
KP270758.1 China
KP270717.1 China
KX788541.1 France
KX788505.1 France
KX788470.1 France
KY495903.1 Italy
PZ121951.1 China
PQ219839.1 China
MT418873.1 France
BJ-08
QN-06
AS-20
GY-16
QXN-11

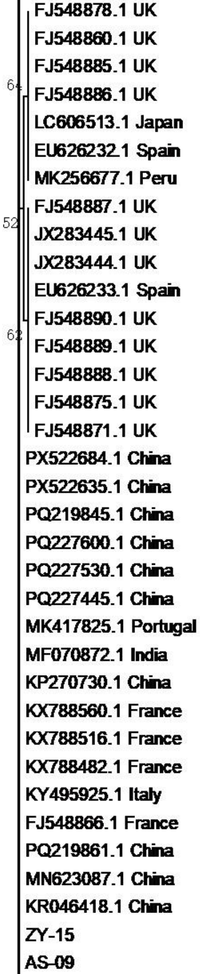
FJ548878.1 UK
FJ548860.1 UK
FJ548885.1 UK
64
FJ548886.1 UK
LC606513.1 Japan
EU626232.1 Spain
MK256677.1 Peru
FJ548887.1 UK
52
JX283445.1 UK
JX283444.1 UK
EU626233.1 Spain
FJ548890.1 UK
62
FJ548889.1 UK
FJ548888.1 UK
FJ548875.1 UK
FJ548871.1 UK
PX522684.1 China
PX522635.1 China
PQ219845.1 China
PQ227600.1 China
PQ227530.1 China
PQ227445.1 China
MK417825.1 Portugal
MF070872.1 India
KP270730.1 China
KX788560.1 France
KX788516.1 France
KX788482.1 France
KY495925.1 Italy
FJ548866.1 France
PQ219861.1 China
MN623087.1 China
KR046418.1 China
ZY-15
AS-09

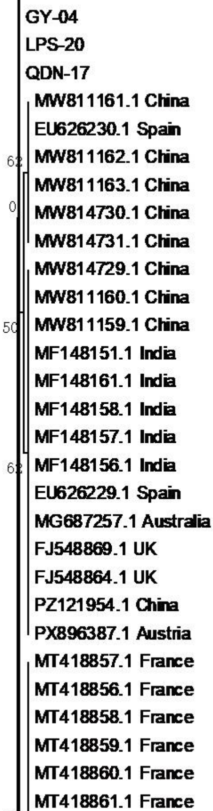
GY-04
LPS-20
QDN-17
MW811161.1 China
EU626230.1 Spain
62
MW811162.1 China
MW811163.1 China
0
MW814730.1 China
MW814731.1 China
MW814729.1 China
MW811160.1 China
50
MW811159.1 China
MF148151.1 India
MF148161.1 India
MF148158.1 India
MF148157.1 India
62
MF148156.1 India
EU626229.1 Spain
MG687257.1 Australia
FJ548869.1 UK
FJ548864.1 UK
PZ121954.1 China
PX896387.1 Austria
MT418857.1 France
MT418856.1 France
MT418858.1 France
MT418859.1 France
MT418860.1 France
MT418861.1 France

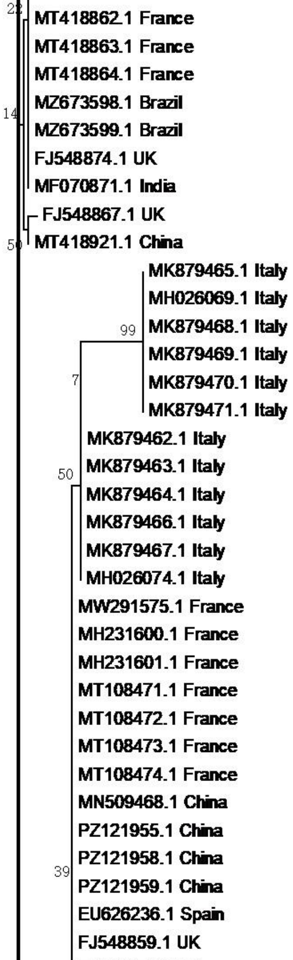

22
MT418862.1 France
MT418863.1 France
MT418864.1 France
14
MZ673598.1 Brazil
MZ673599.1 Brazil
FJ548874.1 UK
MF070871.1 India
FJ548867.1 UK
50
MT418921.1 China
MK879465.1 Italy
MH026069.1 Italy
99
MK879468.1 Italy
MK879469.1 Italy
7
MK879470.1 Italy
MK879471.1 Italy
MK879462.1 Italy
MK879463.1 Italy
50
MK879464.1 Italy
MK879466.1 Italy
MK879467.1 Italy
MH026074.1 Italy
MW291575.1 France
MH231600.1 France
MH231601.1 France
MT108471.1 France
MT108472.1 France
MT108473.1 France
MT108474.1 France
MN509468.1 China
PZ121955.1 China
PZ121958.1 China
39
PZ121959.1 China
EU626236.1 Spain
FJ548859.1 UK

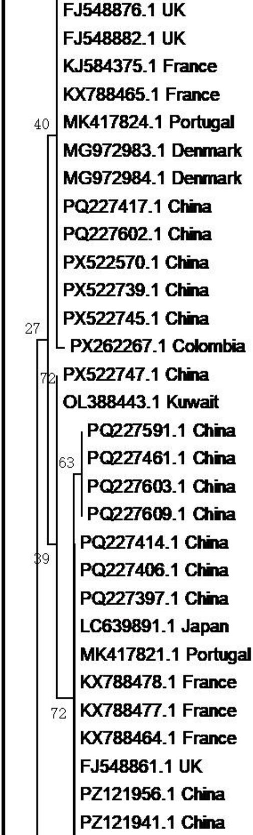
FJ548876.1 UK
FJ548882.1 UK
KJ584375.1 France
KX788465.1 France
40
MK417824.1 Portugal
MG972983.1 Denmark
MG972984.1 Denmark
PQ227417.1 China
PQ227602.1 China
PX522570.1 China
PX522739.1 China
PX522745.1 China
27
PX262267.1 Colombia
72
PX522747.1 China
OL388443.1 Kuwait
PQ227591.1 China
63
PQ227461.1 China
PQ227603.1 China
PQ227609.1 China
PQ227414.1 China
39
PQ227406.1 China
PQ227397.1 China
LC639891.1 Japan
MK417821.1 Portugal
KX788478.1 France
72
KX788477.1 France
KX788464.1 France
FJ548861.1 UK
PZ121956.1 China
PZ121941.1 China

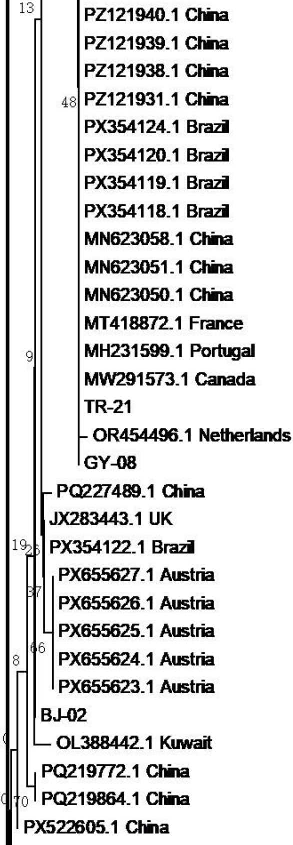
13
PZ121940.1 China
PZ121939.1 China
PZ121938.1 China
48
PZ121931.1 China
PX354124.1 Brazil
PX354120.1 Brazil
PX354119.1 Brazil
PX354118.1 Brazil
MN623058.1 China
MN623051.1 China
MN623050.1 China
MT418872.1 France
MH231599.1 Portugal
9
MW291573.1 Canada
TR-21
OR454496.1 Netherlands
GY-08
PQ227489.1 China
JX283443.1 UK
19
26
PX354122.1 Brazil
PX655627.1 Austria
37
PX655626.1 Austria
PX655625.1 Austria
66
8
PX655624.1 Austria
PX655623.1 Austria
BJ-02
OL388442.1 Kuwait
PQ219772.1 China
70
PQ219864.1 China
PX522605.1 China

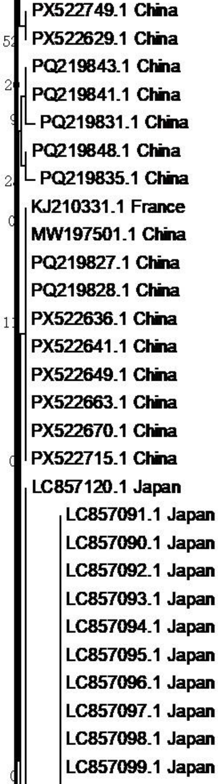
PX522749.1 China
PX522629.1 China
PQ219843.1 China
PQ219841.1 China
PQ219831.1 China
PQ219848.1 China
PQ219835.1 China
KJ210331.1 France
MW197501.1 China
PQ219827.1 China
PQ219828.1 China
PX522636.1 China
PX522641.1 China
PX522649.1 China
PX522663.1 China
PX522670.1 China
PX522715.1 China
LC857120.1 Japan
LC857091.1 Japan
LC857090.1 Japan
LC857092.1 Japan
LC857093.1 Japan
LC857094.1 Japan
LC857095.1 Japan
LC857096.1 Japan
LC857097.1 Japan
LC857098.1 Japan
LC857099.1 Japan

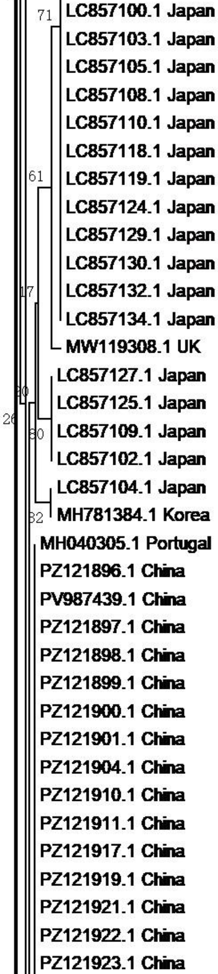
71
LC857100.1 Japan
LC857103.1 Japan
LC857105.1 Japan
LC857108.1 Japan
LC857110.1 Japan
LC857118.1 Japan
61
LC857119.1 Japan
LC857124.1 Japan
LC857129.1 Japan
LC857130.1 Japan
LC857132.1 Japan
LC857134.1 Japan
MW119308.1 UK
LC857127.1 Japan
LC857125.1 Japan
26
LC857109.1 Japan
LC857102.1 Japan
LC857104.1 Japan
MH781384.1 Korea
MH040305.1 Portugal
PZ121896.1 China
PV987439.1 China
PZ121897.1 China
PZ121898.1 China
PZ121899.1 China
PZ121900.1 China
PZ121901.1 China
PZ121904.1 China
PZ121910.1 China
PZ121911.1 China
PZ121917.1 China
PZ121919.1 China
PZ121921.1 China
PZ121922.1 China
PZ121923.1 China

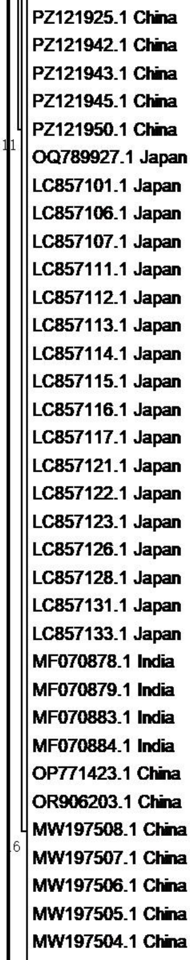
PZ121925.1 China
PZ121942.1 China
PZ121943.1 China
PZ121945.1 China
PZ121950.1 China
OQ789927.1 Japan
LC857101.1 Japan
LC857106.1 Japan
LC857107.1 Japan
LC857111.1 Japan
LC857112.1 Japan
LC857113.1 Japan
LC857114.1 Japan
LC857115.1 Japan
LC857116.1 Japan
LC857117.1 Japan
LC857121.1 Japan
LC857122.1 Japan
LC857123.1 Japan
LC857126.1 Japan
LC857128.1 Japan
LC857131.1 Japan
LC857133.1 Japan
MF070878.1 India
MF070879.1 India
MF070883.1 India
MF070884.1 India
OP771423.1 China
OR906203.1 China
MW197508.1 China
MW197507.1 China
MW197506.1 China
MW197505.1 China
MW197504.1 China

MN623086.1 China
MN623084.1 China
MN623083.1 China
MN623082.1 China
MN623081.1 China
MN623080.1 China
MN623079.1 China
MN623077.1 China
MN623074.1 China
MN623072.1 China
MN623071.1 China
MN623062.1 China
MN623061.1 China
MN623060.1 China
MN623057.1 China
MN623056.1 China
MN623055.1 China
MN623054.1 China
MN623053.1 China
MN623052.1 China
MN623049.1 China
MN623048.1 China
MN623044.1 China
MN623043.1 China
MN623035.1 China
MN623032.1 China
MN623029.1 China
MN623028.1 China
MN623027.1 China
MN623025.1 China
MN623020.1 China
MN623018.1 China
MN623017.1 China
MN623016.1 China
MN623011.1 China

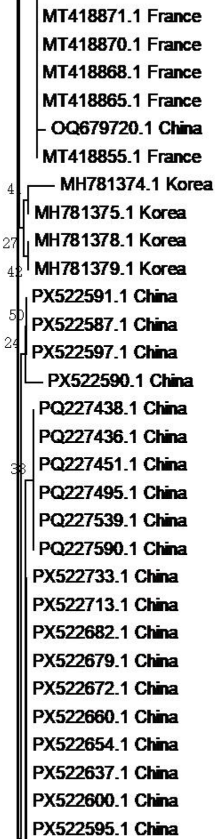

MT418871.1 France
MT418870.1 France
MT418868.1 France
MT418865.1 France
OQ679720.1 China
MT418855.1 France
41
MH781374.1 Korea
MH781375.1 Korea
27
MH781378.1 Korea
42
MH781379.1 Korea
PX522591.1 China
50
PX522587.1 China
24
PX522597.1 China
PX522590.1 China
PQ227438.1 China
PQ227436.1 China
38
PQ227451.1 China
PQ227495.1 China
PQ227539.1 China
PQ227590.1 China
PX522733.1 China
PX522713.1 China
PX522682.1 China
PX522679.1 China
PX522672.1 China
PX522660.1 China
PX522654.1 China
PX522637.1 China
PX522600.1 China
PX522595.1 China

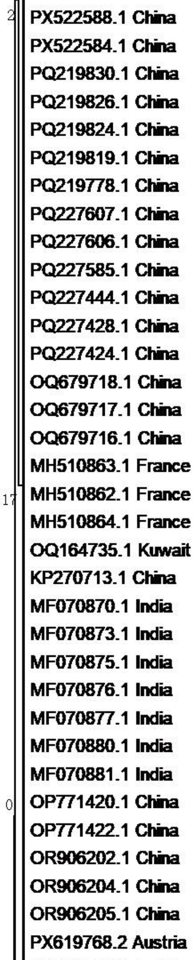
2
PX522588.1 China
PX522584.1 China
PQ219830.1 China
PQ219826.1 China
PQ219824.1 China
PQ219819.1 China
PQ219778.1 China
PQ227607.1 China
PQ227606.1 China
PQ227585.1 China
PQ227444.1 China
PQ227428.1 China
PQ227424.1 China
OQ679718.1 China
OQ679717.1 China
OQ679716.1 China
MH510863.1 France
17
MH510862.1 France
MH510864.1 France
OQ164735.1 Kuwait
KP270713.1 China
MF070870.1 India
MF070873.1 India
MF070875.1 India
MF070876.1 India
MF070877.1 India
MF070880.1 India
MF070881.1 India
0
OP771420.1 China
OP771422.1 China
OR906202.1 China
OR906204.1 China
OR906205.1 China
PX619768.2 Austria

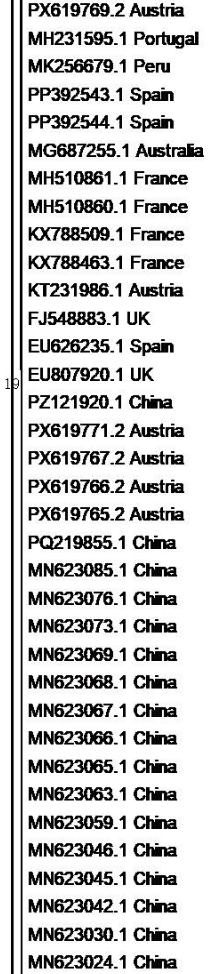
PX619769.2 Austria
MH231595.1 Portugal
MK256679.1 Peru
PP392543.1 Spain
PP392544.1 Spain
MG687255.1 Australia
MH510861.1 France
MH510860.1 France
KX788509.1 France
KX788463.1 France
KT231986.1 Austria
FJ548883.1 UK
EU626235.1 Spain
19
EU807920.1 UK
PZ121920.1 China
PX619771.2 Austria
PX619767.2 Austria
PX619766.2 Austria
PX619765.2 Austria
PQ219855.1 China
MN623085.1 China
MN623076.1 China
MN623073.1 China
MN623069.1 China
MN623068.1 China
MN623067.1 China
MN623066.1 China
MN623065.1 China
MN623063.1 China
MN623059.1 China
MN623046.1 China
MN623045.1 China
MN623042.1 China
MN623030.1 China
MN623024.1 China

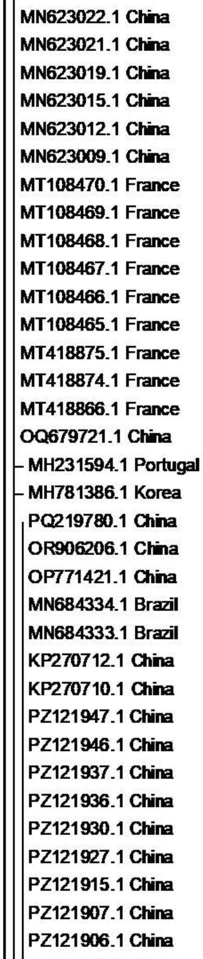
MN623022.1 China
MN623021.1 China
MN623019.1 China
MN623015.1 China
MN623012.1 China
MN623009.1 China
MT108470.1 France
MT108469.1 France
MT108468.1 France
MT108467.1 France
MT108466.1 France
MT108465.1 France
MT418875.1 France
MT418874.1 France
MT418866.1 France
OQ679721.1 China
MH231594.1 Portugal
MH781386.1 Korea
PQ219780.1 China
OR906206.1 China
OP771421.1 China
MN684334.1 Brazil
MN684333.1 Brazil
KP270712.1 China
KP270710.1 China
PZ121947.1 China
PZ121946.1 China
PZ121937.1 China
PZ121936.1 China
PZ121930.1 China
PZ121927.1 China
PZ121915.1 China
PZ121907.1 China
PZ121906.1 China

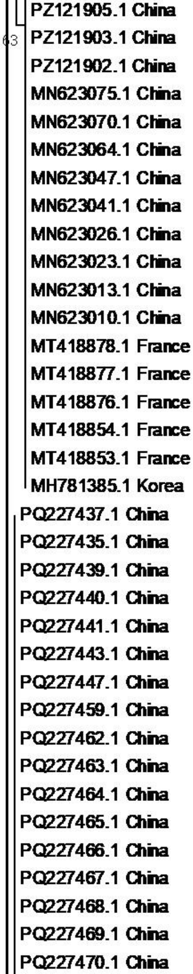
PZ121905.1 China
63
PZ121903.1 China
PZ121902.1 China
MN623075.1 China
MN623070.1 China
MN623064.1 China
MN623047.1 China
MN623041.1 China
MN623026.1 China
MN623023.1 China
MN623013.1 China
MN623010.1 China
MT418878.1 France
MT418877.1 France
MT418876.1 France
MT418854.1 France
MT418853.1 France
MH781385.1 Korea
PQ227437.1 China
PQ227435.1 China
PQ227439.1 China
PQ227440.1 China
PQ227441.1 China
PQ227443.1 China
PQ227447.1 China
PQ227459.1 China
PQ227462.1 China
PQ227463.1 China
PQ227464.1 China
PQ227465.1 China
PQ227466.1 China
PQ227467.1 China
PQ227468.1 China
PQ227469.1 China
PQ227470.1 China

PQ227471.1 China
PQ227472.1 China
PQ227473.1 China
PQ227474.1 China
PQ227475.1 China
PQ227476.1 China
PQ227477.1 China
PQ227478.1 China
PQ227479.1 China
PQ227480.1 China
PQ227481.1 China
PQ227482.1 China
PQ227483.1 China
PQ227487.1 China
PQ227488.1 China
PQ227490.1 China
PQ227494.1 China
PQ227516.1 China
PQ227522.1 China
PQ227534.1 China
PQ227536.1 China
PQ227537.1 China
PQ227538.1 China
PQ227540.1 China
PQ227544.1 China
PQ227546.1 China
PQ227548.1 China
PQ227549.1 China
PQ227550.1 China
PQ227552.1 China
PQ227553.1 China
PQ227554.1 China
PQ227556.1 China
PQ227557.1 China

24

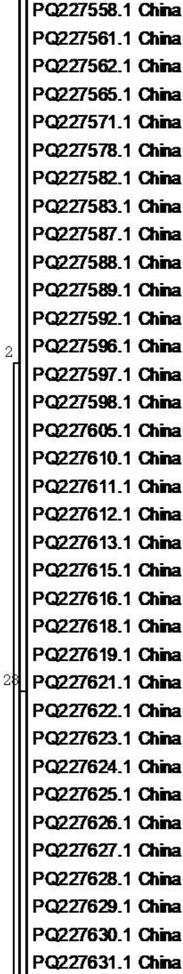
PQ227558.1 China
PQ227561.1 China
PQ227562.1 China
PQ227565.1 China
PQ227571.1 China
PQ227578.1 China
PQ227582.1 China
PQ227583.1 China
PQ227587.1 China
PQ227588.1 China
PQ227589.1 China
PQ227592.1 China
2
PQ227596.1 China
PQ227597.1 China
PQ227598.1 China
PQ227605.1 China
PQ227610.1 China
PQ227611.1 China
PQ227612.1 China
PQ227613.1 China
PQ227615.1 China
PQ227616.1 China
PQ227618.1 China
PQ227619.1 China
PQ227621.1 China
PQ227622.1 China
PQ227623.1 China
PQ227624.1 China
PQ227625.1 China
PQ227626.1 China
PQ227627.1 China
PQ227628.1 China
PQ227629.1 China
PQ227630.1 China
PQ227631.1 China

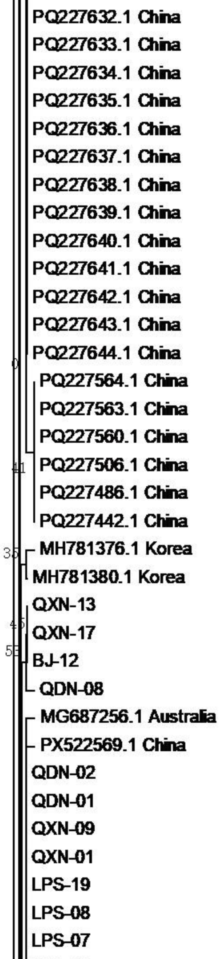
PQ227632.1 China
PQ227633.1 China
PQ227634.1 China
PQ227635.1 China
PQ227636.1 China
PQ227637.1 China
PQ227638.1 China
PQ227639.1 China
PQ227640.1 China
PQ227641.1 China
PQ227642.1 China
PQ227643.1 China
PQ227644.1 China
PQ227564.1 China
PQ227563.1 China
PQ227560.1 China
PQ227506.1 China
PQ227486.1 China
PQ227442.1 China
MH781376.1 Korea
MH781380.1 Korea
QXN-13
QXN-17
BJ-12
QDN-08
MG687256.1 Australia
PX522569.1 China
QDN-02
QDN-01
QXN-09
QXN-01
LPS-19
LPS-08
LPS-07

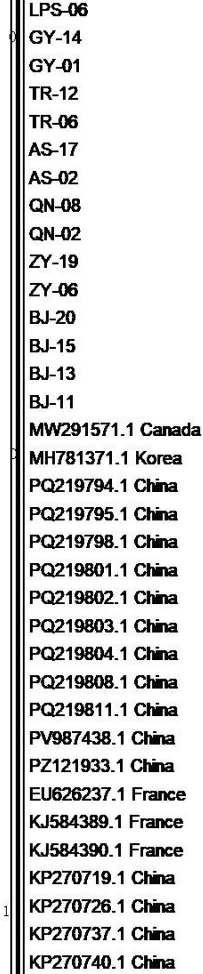
LPS-06
GY-14
GY-01
TR-12
TR-06
AS-17
AS-02
QN-08
QN-02
ZY-19
ZY-06
BJ-20
BJ-15
BJ-13
BJ-11
MW291571.1 Canada
MH781371.1 Korea
PQ219794.1 China
PQ219795.1 China
PQ219798.1 China
PQ219801.1 China
PQ219802.1 China
PQ219803.1 China
PQ219804.1 China
PQ219808.1 China
PQ219811.1 China
PV987438.1 China
PZ121933.1 China
EU626237.1 France
KJ584389.1 France
KJ584390.1 France
KP270719.1 China
KP270726.1 China
KP270737.1 China
KP270740.1 China

KP270746.1 China
KP270752.1 China
PX522755.1 China
PX522754.1 China
PX522752.1 China
PX522742.1 China
PX522731.1 China
PX522730.1 China
PX522723.1 China
PX522721.1 China
PX522639.1 China
PX522628.1 China
PX522613.1 China
PX522610.1 China
PX522601.1 China
PX522582.1 China
PX522576.1 China
PQ219791.1 China
PQ219790.1 China
PQ219787.1 China
PQ219782.1 China
PQ227579.1 China
PQ227529.1 China
PQ227527.1 China
PQ227525.1 China
PQ227519.1 China
PQ227518.1 China
PQ227514.1 China
PQ227508.1 China
PQ227505.1 China
PQ227504.1 China
PQ227493.1 China
PQ227492.1 China
PQ227429.1 China

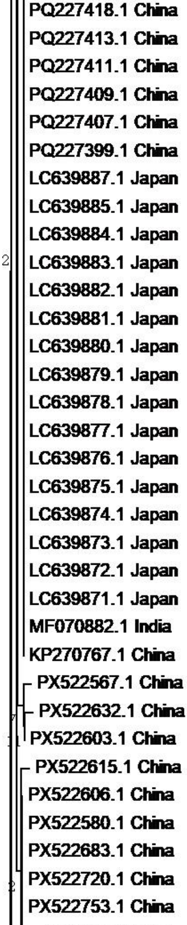
PQ227418.1 China
PQ227413.1 China
PQ227411.1 China
PQ227409.1 China
PQ227407.1 China
PQ227399.1 China
LC639887.1 Japan
LC639885.1 Japan
LC639884.1 Japan
2
LC639883.1 Japan
LC639882.1 Japan
LC639881.1 Japan
LC639880.1 Japan
LC639879.1 Japan
LC639878.1 Japan
LC639877.1 Japan
LC639876.1 Japan
LC639875.1 Japan
LC639874.1 Japan
LC639873.1 Japan
LC639872.1 Japan
LC639871.1 Japan
MF070882.1 India
KP270767.1 China
PX522567.1 China
PX522632.1 China
PX522603.1 China
PX522615.1 China
PX522606.1 China
PX522580.1 China
PX522683.1 China
PX522720.1 China
PX522753.1 China

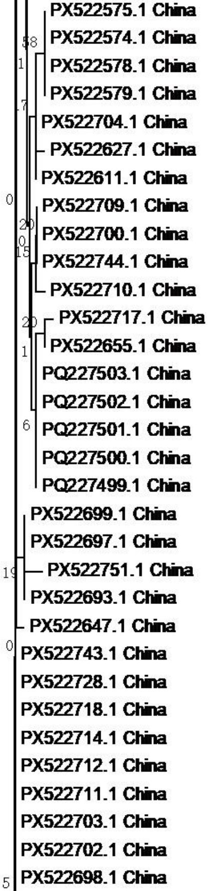
PX522575.1 China
58
PX522574.1 China
1
PX522578.1 China
PX522579.1 China
PX522704.1 China
PX522627.1 China
PX522611.1 China
0
PX522709.1 China
20
PX522700.1 China
0
15
PX522744.1 China
PX522710.1 China
20
PX522717.1 China
1
PX522655.1 China
PQ227503.1 China
PQ227502.1 China
6
PQ227501.1 China
PQ227500.1 China
PQ227499.1 China
PX522699.1 China
PX522697.1 China
19
PX522751.1 China
PX522693.1 China
PX522647.1 China
0
PX522743.1 China
PX522728.1 China
PX522718.1 China
PX522714.1 China
PX522712.1 China
PX522711.1 China
PX522703.1 China
PX522702.1 China
5
PX522698.1 China

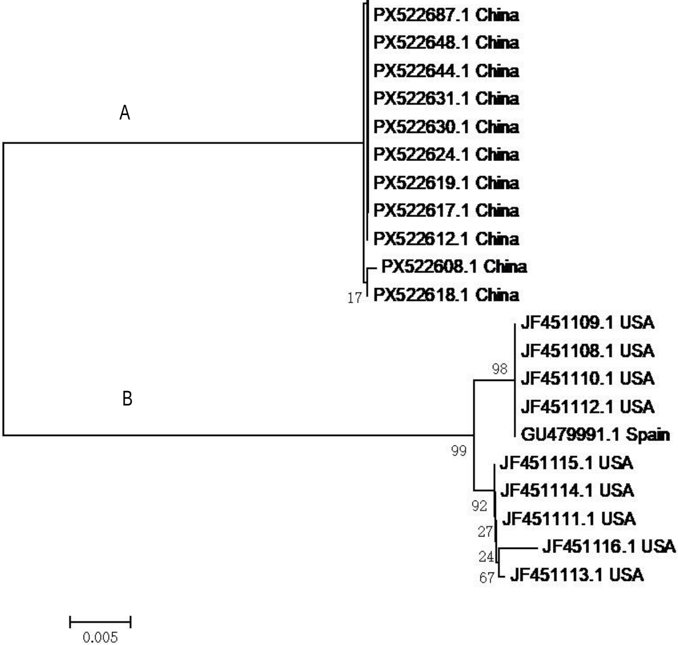
A
PX522687.1 China
PX522648.1 China
PX522644.1 China
PX522631.1 China
PX522630.1 China
PX522624.1 China
PX522619.1 China
PX522617.1 China
PX522612.1 China
PX522608.1 China
17
PX522618.1 China
B
JF451109.1 USA
JF451108.1 USA
98
JF451110.1 USA
JF451112.1 USA
GU479991.1 Spain
99
JF451115.1 USA
JF451114.1 USA
92
JF451111.1 USA
27
JF451116.1 USA
24
67
JF451113.1 USA
0.005
