# Supplementary material for: Antifungal Susceptibility and Cyp51A Gene Variation Analysis of Aspergillus fumigatus Isolated from Soils in Tea-Growing Areas of Guizhou, China
Source: Microorganisms. 2026 Aug 4;14(8):1704. doi: 10.3390/microorganisms14081704 (PMC13515310; doi:10.3390/microorganisms14081704)
Supplement: Supplementary file 1 [file microorganisms-14-01704-s001.zip › Table S1. Information on variant sites of the cyp51A gene CDS in Aspergillus fumigatus..pdf]

Table S1. Information on variant sites of the *cyp51A* gene CDS in *A. fumigatus.*

Cyp51A CDS mutation sites	Nucleotide substitution	Number of substitutions	Frequency	Variant type
137	T→A	2	0.0105	Nonsynonymous
267	G→A	3	0.0157	Synonymous
514	A→G	4	0.0209	Nonsynonymous
540	G→A	4	0.0209	Synonymous
743	A→C	2	0.0105	Nonsynonymous
744	T→A	23	0.1204	Nonsynonymous
765	C→G	2	0.0105	Nonsynonymous
1074	A→G	3	0.0157	Synonymous
1279	G→A	2	0.0105	Nonsynonymous
1362	T→C	3	0.0157	Synonymous
